# 

**Published:** 1896-11

**Authors:** 


### CONTENTS.

PROCEEDINGS.

Points of Interest in the Care of Children's Teeth..... .. ................ 521

Personal Observations of the Development of Dentistry and Dental Legislation in Michigan.............................................. 548

MONTHLY DIGEST.

Dental Amalgams...................... .......................................... 568

SELECTIONS.

Dr. Jack's Views on Implantation................................................ 572

EDITORIAL.

The National Association of Dental Faculties.................. .......... 569

Bibliographical .......................................................................... 570

Ohio State Dental Society.............. ..... ........ ....................... 571

